# The Effects of Reducing Vehicular Emissions in Atmospheric Hydrocarbon Concentrations in Rio de Janeiro, Brazil

**DOI:** 10.1021/acsomega.5c07885

**Published:** 2025-11-14

**Authors:** Margarida Maria Sartori Tavares, Rennan Guedes Carneiro, Graciela Arbilla, Cleyton Martins da Silva, Sergio Machado Corrêa

**Affiliations:** † 663957 Universidade do Estado do Rio de Janeiro, Resende, Rio de Janeiro 27537-000, Brazil; ‡ 28125 Universidade Federal do Rio de Janeiro, Instituto de Química, Rio de Janeiro, Rio de Janeiro 21941-909, Brazil; § 186094 Universidade Veiga de Almeida, Campus Tijuca, Rio de Janeiro, Rio de Janeiro 20271-020, Brazil

## Abstract

Vehicles in urban environments are the main source of criteria pollutants as well as hydrocarbons (HC), which contribute to the formation of ozone and secondary organic aerosols (especially fine particles, PM_2.5_). In Brazil, HC emission standards have been set by the National Program for Controlling Air Pollution by Vehicles (PROCONVE) since 2007. The purpose of this study was to determine the concentration, speciation, and ozone forming potential (OFP) of C_2_–C_12_ HC, collected, in 2023, in an urban area of the city of Rio de Janeiro, that is characterized by vehicular emissions. Samples were collected by means of stainless-steel canisters, and then thermally desorbed and analyzed by heart-cutting multidimensional gas chromatography, with the aid of two detectors (MSD and FID) and two columns. The results were compared with data published in previous years, in the same seasonal period (the dry season) and at locations with similar emission features, to assess the effectiveness of the Brazilian vehicular emission control system. They show a reduction of total concentrations, in particular of aromatic compounds, and a lowering of the average OFP, despite an increase in the traffic volume. Clearly, the reduction in concentrations and atmospheric reactivity has resulted from attempts to ensure the vehicular fleet meets the requirements of PROCONVE.

## Graphical abstract

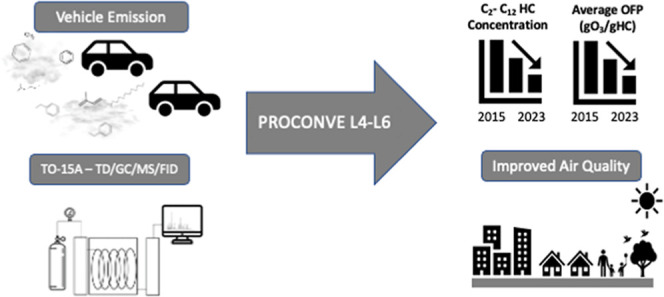

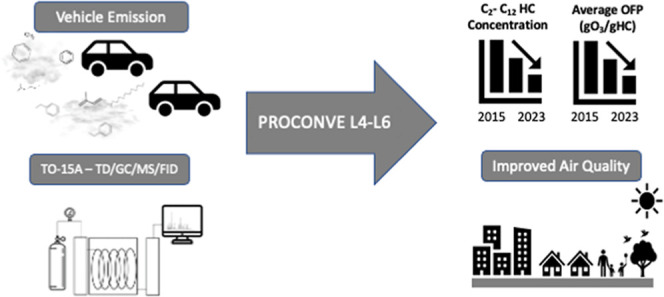

## Introduction

Vehicle emissions are the main source of hydrocarbons (HC) in urban environments which contribute to the formation of ozone and secondary aerosols (especially fine particles, PM_2.5_) that are currently the criteria pollutants of main concern.^1^ In Brazil, these emissions have some particularities related to the use of certain fuels: H100 (100% hydrous ethanol), gasohol (gasoline blended with 18–27% v/v anhydrous ethanol), compressed natural gas, and diesel (with 14% biodiesel). According to an official report published by the Ministry of Environment, it was estimated that approximately 71% of nonmethane hydrocarbons (NMHC) are caused by light-duty vehicles and motorcycle emissions.^2^ Flex-fuel vehicles, which represent more than 80% of the Brazilian fleet of light-duty vehicles, can operate with H100 and gasohol.^3^ Motorcycles also run on gasohol.

Brazilian vehicular emission standards are determined by the Ministry of Environment through the National Program for Controlling Air Pollution by Vehicles (PROCONVE). Since 1988, emission standards have been revised and updated through several PROCONVE-L phases (PROCONVE for light-duty vehicles, up to 3856 kg). In 2015, Carvalho et al.^4^ evaluated the pollutant concentrations that had been measured by the air quality monitoring network of the Metropolitan Area of São Paulo (MASP), in the period 1996–2009, and found that despite the increase in the number of vehicles, there was a reduction in the total emissions of regulated pollutants. The authors concluded that emission control policies played a key role in the reduction of all criterion pollutants, except ozone.

With regard to NMHC emissions, limits were established by CONAMA^5,6^ with effect from January 2007. For PROCONVE L4 vehicles (2007–2008), the emission limit was 0.16 g km^–1^, while for L5 (2009–2012) and L6 (2013–2021) vehicles, it was 0.05 g km^–1^.^5,7,8^ Recently, three flex-fuel vehicles (PROCONVE L4, L5, and L6) were tested by means of a chassis dynamometer, in accordance with Brazilian standards^8^ and E22 (gasoline blended with 22% v/v anhydrous ethanol). Total emissions were 0.047, 0.025, and 0.0042 g km^–1^ for the L4, L5, and L6 vehicles respectively, which showed that their performance was much better than had been expected, considering the PROCONVE maximum values.^3,9^ In a previous study, a C_2_–C_12_ HC emission factor of 0.065 g km^–1^ was measured for a PROCONVE L3 vehicle.^10^

Determining C_2_–C_12_ HC concentrations, in both atmospheric and exhaust emission samples, is, generally, a complex task. The U.S. Environmental Protection Agency (U.S. EPA) has drawn up a Quality Assurance Project Plan which covers the operation of Photochemical Assessment Monitoring Stations for determining speciated Volatile Organic Compounds (VOC), both HC and carbonyls, and the meteorological parameters.^11^ The determination of speciated VOC should be conducted continuously by automated gas chromatograph or auto-GC.^12^ In Brazil, automatic air quality monitoring stations do not determine speciated VOC. In general, hydrocarbon and carbonyl compounds are analyzed as described by U.S. EPA in a compendium of 17 standardized methods for determining toxic organic compounds in ambient air.^13^

Current analytical methods have been fully described by Da Silva et al.^14^ and include continuous sampling and collection with canisters and Tedlar bags. In Brazil, continuous sampling has been carried out in both urban and green areas.^15,16^ While sampling with Tedlar bags is currently used for the study of exhaust vehicular emissions,^17^ canisters have been adopted in several situations, as described in the TO-15 and TO-15A methods. These methods rely on gas chromatography (GC) and mass spectrometry and are a highly effective means of determining C_4_–C_12_ compounds, as well as C_3_ HC with subambient cooling.^18^ Heart-cutting multidimensional GC is a further improvement to the TO-15A method.^14^ With the aid of capillary flow technology, Deans Switch, two detectors (MSD and FID), and two columns, C_2_–C_12_ HC can be speciated with a single GC system. The speciation of C_2_ species is a key factor in an ethanol burning scenario, like that in Brazil, where ethane and ethene represent significant parts of the total HC.

The main concern of this study is to evaluate the contribution made by PROCONVE HC emission control policies to the total amount and speciation of C_2_–C_12_ HC, as well as the importance of determining lighter species in an urban environment affected by flex-fuel vehicular emissions. Thus, its objective is to determine the concentration, speciation, and ozone forming potential (OFP) of C_2_–C_12_ HC and compare these results with previously published data, when L4 and L5 vehicles made a greater contribution to total emissions, as a means of assessing the effectiveness of Brazilian vehicular emission control systems.

## Materials and Methods

### Studied Area

The State of Rio de Janeiro (ERJ), in south Brazil, has a population of approximately 17 million inhabitants and 8 million vehicles (approximately 5 million light-duty vehicles and 1.3 million motorcycles).^19^ According to the most recent vehicular emission inventory, in 2022, the total amount of NMHC vehicular emission was 12,989 t. Otto-cycle vehicles and motorcycles contributed 74 and 6.7%, respectively. Regarding nitrogen oxides emissions (NO_*x*_), which also play an important role in ozone balance, 80% is caused by diesel vehicles.^20^

The city of Rio de Janeiro (CRJ) is part of the Metropolitan Region of Rio de Janeiro (MRRJ), the third largest metropolitan area in South America, with 22 municipalities, an area of 8326 km^2^, and a population of approximately 13.5 million inhabitants.^19^ The CRJ is the State capital with a population of 6.7 million inhabitants. It is located on the coast of the Atlantic Ocean and more than 27% of its surface is covered by the preserved Atlantic rainforest,^21^ including the Tijuca Forest (maximum altitude 1022 m), the Pedra Branca park with the Pedra Branca Massif (1024 m), which is the highest point of the city, and the Gericinó-Mendanha park. These urban forests and the ocean play an important role in atmospheric circulation.^1^ The climate is Aw (Atlantic tropical) with a dry season from April to September.

According to the Rio de Janeiro municipal records, in 2023, the total number of vehicles was 3.4 million, approximately 77% of them being light-duty, distributed in 55%, 10%, and 35% in PROCONVE L4, L5, and L6 phases, respectively.^22^

Atmospheric samples were collected using canisters in May-July 2023 at the Maracanã campus of the Rio de Janeiro State University (UERJ), at a distance of about 100 m from Maracanã metro station, close to Maracanã Stadium and Avenue Rei Pele (Radial Oeste). Figure S1 shows a map of the city of Rio de Janeiro and the studied location. The area is characterized by a steady flow of vehicles and people. It is a fully urbanized area, both residential and commercial, with a large number of buildings. Emission sources are vehicular (mainly light-duty vehicles and buses). It is approximately 2.3 km from the automated monitoring station located at Saens Pea Square (District of Tijuca, Avenue Conde de Bonfim and General Roca Street) operated by the Municipal Department of Environment and Climate (SMAC).

### Data Collection and Analysis

Samples were collected with the aid of clean evacuated 6.0 L stainless steel canisters (Entech Silonite and Restek SilcoCan) and flow restrictors (Entech Flow Controller CS1200E) to meet a sampling time of 1 h. Samples were collected between 8:00 and 9:00 h to reduce the photochemical decomposition and the reaction of organic compounds. This is, in general, the sampling time adopted in previous studies that were used for comparison. The cleaning and sampling procedure is fully described in the literature.^23,24^

A total of 12 samples was analyzed by triplicate. C_2_–C_12_ HC determination was conducted as described by Da Silva et al.^14^ All the experimental details of this method, as well as its calibration, control quality, and performance are fully discussed in this previous article.^14^ Typically, an aliquot of 500 mL of the sample was transferred through a Nafion dryer trap (AS3/8 Series 2 Markes International, OH, USA) and preconcentrated in a cold trap (U-T3ATX-2S, Markes International, OH, USA) at −20 °C. The target HC were thermally desorbed at 300 °C and transferred to a GC (Agilent model GC 7890A, CA, USA) with MSD and FID, and a capillary flow technology Deans Switch. The primary column was a DB-624 (60 m × 0.32 mm × 1.8 μm), and the secondary one was a PoraPlot Q-HT (25 m × 0.32 mm × 10 μm). The oven temperature program was set as 27 °C (10 min), 5 °C min^–1^ up to 240 °C, and after 1 min, a postrun at 250 °C. C_2_–C_3_ compounds were resolved using both columns and quantified by FID. C_4_–C_12_ HC were resolved by means of the primary column and quantified by MSD.

A standard reference mixture (57 compounds, Restek, 20–60 ppbc, PO#P2341, PA, USA) was used both to confirm the identity of the individual HC and to construct the individual external analytical curves. The limits of detection (LOD) and quantification (LOQ) were 0.1 and 0.3 ng, respectively, for all the compounds. Thus, the method detection and quantification limits (within the conditions of analysis) were calculated as 0.2 μg m^–3^ and 0.6 μg m^–3^, respectively.

### HC Reactivity and Ozone Forming Potential (OFP)

The OPF of each individual HC was calculated as described previously,^25^ using the Maximum Incremental Reactivity (MIR) scale developed by Carter.^26^ The OFP for each compound (OFP_i_) was evaluated by eq 1

1 $${OFP}_{i}={{MIR}_{i}}^{*}{[HC}_{i}]$$

where [HC_i_] are the individual HC concentrations.

MIR_i_ are the dimensionless IR coefficients for each compound, as estimated by Carter^26^ using the MIR scale.

Then, the average reactivity (average IR in MIR scale) or average OFP, for each individual sample was evaluated by means of eq 2

2 $$Average\;OFP=Average\;IR=\left[\sum \left({MIR}_{i}\times {HC}_{i}\right)\right]/total\;HC\;concentration$$

where MIR_i_ = MIR coefficient for each compound and [HC]_i_ = concentration of each HC in μg m^–3^

## Results and Discussion

### Total HC Concentrations

Total C_2_–C_12_ HC concentrations were in the interval 28.2 to 631.0 μg m^–3^ with a median value of 101.0 μg m^–3^. The median value for the C_4_–C_12_ compounds was 72.7 μg m^–3^. C_2_ compounds represented 12.1% of the total mixture, which confirms the importance of determining these compounds (mainly ethane and ethene) in urban environments, where ethanol and gasoline-ethanol blends are used.

In Table 1, these results are compared with the previously determined values in Rio de Janeiro. In all these studies, the sampling and analysis was based on Method TO-15 ^18^ or on the modified method using heart-cutting multidimensional GC,^14^ the sampling time was 1 h (8:00–9:00 h) and the standard references mixture included the same 57 compounds.

**Table 1:** Total C_2_–C_12_ HC and C_4_–C_12_ HC Median Values Determined in This Study and Literature Data Obtained in Other Locations in the City of Rio de Janeiro

local	date	C_2_–C_3_ HC (μg m^–3^)	C_4_–C_12_ HC (μg m^–3^)	main emission sources	reference
UERJ	May-July 2023 (dry season)	28.3	72.7	vehicular emissions	this study^a^
UERJ	January 2012 (rainy season)	ND	708.5	vehicular emissions	^27^ ^b^
Saens Peña Square (Tijuca)	October 2022-March 2023 (rainy season)	20.0	37.9	vehicular emissions	^25^ ^a^
Del Castilho		42.6	106.0	vehicular emissions and transport of industrial emissions	
Bangu	July 2016 (dry season)	ND	112.6	vehicular emissions	^28^ ^b^
Saens Peña Square (Tijuca)	March 2015 (rainy season)	ND	140.2	vehicular emissions	^24^ ^b^
	May-June 2015 (dry season)	ND	333.0		
	August 2015 (dry season)	ND	225.0		
	November 2015 (rainy season)	ND	191.5		

a Notes: values obtained using the method described in this study.

b Values obtained using the TO-15 method (TD/GC/MSD with a DB-624 column),^18^ ND: not determined (because the authors used the original method TO-15 or TO-15A).

As noted previously, the distance between the UERJ (Maracanã campus) and Saens Pea Square is approximately 2.3 km. Although the vehicular fleet profile is similar, the number of vehicles is at least twice as large at the UERJ campus,^29,30^ which explains why the results obtained at the Saens Peña Square in 2022–2023 were lower than the values determined in this study.

Although the C_4_–C_12_ HC total values obtained at Saens Peña Square in 2015 were higher in all the sampling campaigns than the median value for 2023 (72.7 μg m^–3^), this difference is higher when the results for May-June 2013 are compared. These values are similar to that obtained at Saens Peña Square (74.6 μg m^–3^) during the 2016 Summer Olympic Games when the flow of light vehicles was reduced on account of driving restrictions imposed in the Olympic areas (such as Maracanã), and holidays in schools, universities, and public offices.^31^ In all of the campaigns, samples were collected from 8:00 to 9:00 h (when the photochemical activity is lower). Hence, it can be assumed that the lower values obtained during the Olympic Games and in this study are due to a reduction in vehicular emissions. In August 2016, this decrease was the consequence of the restrictions in vehicular flow, and in 2023, it can be attributed to the improvement in the vehicular fleet, particularly with the introduction of PROCONVE L6.

### Individual C_2_–C_12_ HC Speciation and Reactivity

All the 57 compounds of the reference mixture were analyzed but concentrations of *cis*-2-butene, 2,2,4-trimethylpentane, 2,3,4-trimethylpentane, 3-methylheptane, styrene, cumene, *n*-propylbenzene, m-, p- and *o*-ethyltoluene, 1,3,5-trimethylbenzene, 1,3 and 1,4-diethylbenzene, undecane, and dodecane were < LOD in all the samples.

The median concentrations of 20 compounds were > LOQ (0.6 μg m^–3^). The 15 most abundant compounds (*n*-butane, propane, isopentane, ethane, *n*-pentane, isobutane, 2-methylpentane, *n*-hexane, toluene, 2,3-dimethlylbutane, 1-butene, *n*-heptane, propene, methylcyclopentane, and 3-methylpentane), represent 94% of total mass and are shown in Figure 1. The other compounds with median > LOQ are ethylene, isoprene (2-methyl-1,3-butadiene), 2-methylhexane, benzene, and (m + p)-xylenes.

**Figure 1:**
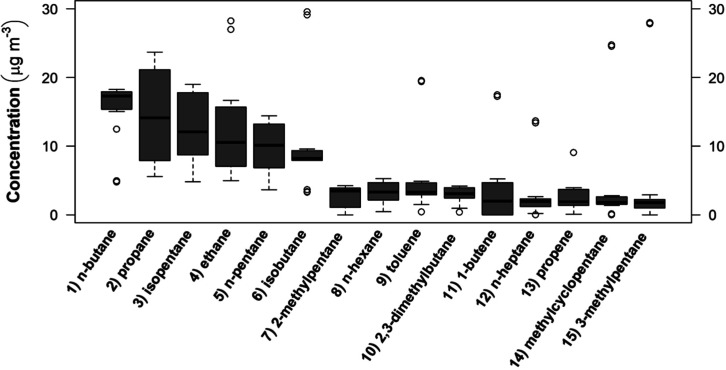
Concentrations (μg m^–3^) of the top-15 most abundant C_2_–C_12_ compounds determined at the UERJ campus in May-July 2023.

All these compounds were also the most abundant HC that were determined in an underground parking lot^14^ which confirms that they are related to emissions from light-duty vehicles. In contrast, at Irajá, a District in the northern area of the city, with mixed emission sources and a significant contribution through the air masses that are transported from the northern industrial zone of the MRRJ, the most abundant C_4_–C_12_ HC were isobutane, isopentane, *n*-butane, *n*-pentane, dodecane, *n*-nonane, toluene, 1,2,3-trimethylbenzene, (m + p)-xylenes, 1,3,5-trimethylbenzene, 1,4-diethylbenzene, 1,3-diethylbenzene, *n*-hexane, *n*-propylbenzene, and *o*-ethyltoluene.^32^ Furthermore, the amount of aromatic compounds at Irajá was 43.3%, which shows a different profile with regard to speciation and the importance of industrial emissions in this part of the city.

In Table 2, the median values of the individual HC determined in this study and at Saens Pea Square in May-June 2015 are shown for purposes of comparison (only values ≥ LOQ are shown). These data were selected for comparison because they were obtained during the same season at a location with similar features.

**Table 2:** Median, Minimum, and Maximum Concentration Values for the Determined HC in This Study (May-July 2023) and at Sans Peña Square (May-June 2015) (Values in μg m^–3^)­^a^

	UERJ, Maracanã (May-July 2023)^b^			Saens Peña Square, Tijuca (May-June 2015)^c^		
compounds	median	minimum	maximum	median	minimum	maximum
ethylene	1.7	0.1	11.1	ND	ND	ND
acetylene	<LOQ	<LOQ	1.7	ND	ND	ND
ethane	10.5	5.0	37.4	ND	ND	ND
propane	14.1	5.6	41.5	ND	ND	ND
propene	1.9	0.1	9.1	ND	ND	ND
isobutane	8.2	3.3	29.6	43.0	23.5	63.3
1-butene	2.0	<LOQ	17.5	23.3	12.2	29.4
*n*-butane	17.3	4.8	73.0	68.0	37.4	104.0
*trans*-2-butene	<LOQ	<LOQ	0.4	4.4	1.7	5.5
*cis*-2-butene	<LOQ	<LOQ	<LOQ	2.0	1.1	2.7
isopentane	12.1	4.8	19.0	39.8	10.9	74.3
1-pentene	<LOQ	<LOQ	10.6	1.6	1.5	1.7
*n*-pentane	10.1	3.6	14.4	39.7	8.1	73.0
*cis*-2-pentene	<LOQ	<LOQ	3.4	2.3	0.9	4.1
isoprene	0.9	<LOQ	2.1	2.1	1.2	2.9
*trans*-2-pentene	<LOQ	<LOQ	1.0	0.8	0.7	1.0
2,2-dimethylbutane	<LOQ	<LOQ	8.7	1.5	1.3	1.7
2,3-dimethylbutane	3.1	0.4	41.6	2.9	2.7	3.0
2-methylpentane	3.5	<LOQ	56.3	5.6	1.7	9.5
cyclopentane	<LOQ	<LOQ	17.0	<LOQ	<LOQ	<LOQ
3-methylpentane	1.8	<LOQ	28.0	4.6	1.1	8.4
1-hexene	<LOQ	<LOQ	3.8	<LOQ	<LOQ	<LOQ
*n*-hexane	3.3	0.4	49.8	15.1	3.2	30.5
2,4-dimethylpentane	<LOQ	<LOQ	3.6	0.8	0.8	0.8
methylcyclopentane	1.8	<LOQ	24.7	3.1	1.1	5.9
2-methylhexane	1.3	<LOQ	12.6	3.4	0.9	3.9
cyclohexane	<LOQ	<LOQ	18.3	3.2	3.2	3.3
2,3 dimethylpentane	<LOQ	<LOQ	6.3	1.1	1.0	1.2
3-methylhexane	<LOQ	<LOQ	12.8	4.8	4.5	5.1
benzene	1.6	<LOQ	10.0	5.2	1.9	7.5
2,2,4-trimethylpentane	<LOQ	<LOQ	<LOQ	0.4	0.4	0.5
*n*-heptane	1.9	<LOQ	13.7	4.2	2.3	7.5
methylcyclohexane	<LOQ	<LOQ	1.4	2.6	0.8	5.4
2,3,4-trimethylpentane	<LOQ	<LOQ	<LOQ	<LOQ	<LOQ	<LOQ
2-methylheptane	<LOQ	<LOQ	0.8	1.6	1.4	1.7
3-methylheptane	<LOQ	<LOQ	<LOQ	1.8	1.6	1.9
toluene	3.3	<LOQ	19.6	17.3	5.1	29.2
*n*-octane	<LOQ	<LOQ	4.5	1.2	1.0	3.5
ethylbenzene	<LOQ	<LOQ	2.4	2.5	0.6	4.2
(m + p)-xylenes	0.7	<LOQ	3.5	2.8	1.0	4.5
*n*-nonane	<LOQ	<LOQ	0.58	2.2	1.3	3.1
o-xylene	<LOQ	<LOQ	1.5	2.2	0.7	3.8
styrene	<LOQ	<LOQ	<LOQ	1.1	1.1	1.2
cumene	<LOQ	<LOQ	<LOQ	<LOQ	<LOQ	<LOQ
*n*-propylbenzene	<LOQ	<LOQ	<LOQ	0.7	<LOQ	0.7
(m + p)-ethyltoluenes	<LOQ	<LOQ	<LOQ	2.9	1.0	3.0
*n*-decane	<LOQ	<LOQ	2.1	1.9	1.0	5.20
1,3,5-trimethylbenzene	<LOQ	<LOQ	<LOQ	0.7	0.7	0.7
*o*-ethyltoluene	<LOQ	<LOQ	<LOQ	<LOQ	<LOQ	<LOQ
1,2,4-trimethylbenzene	<LOQ	<LOQ	2.8	2.5	1.0	3.9
1,2,3-trimethylbenzene	<LOQ	<LOQ	0.6	0.7	0.7	0.7
*m*-diethylbenzene	<LOQ	<LOQ	<LOQ	<LOQ	<LOQ	<LOQ
*p*-diethylbenzene	<LOQ	<LOQ	<LOQ	<LOQ	<LOQ	<LOQ
*n*-undecane	<LOQ	<LOQ	<LOQ	2.3	0.6	2.5
*n*-dodecane	<LOQ	<LOQ	<LOQ	2.1	2.1	2.1

a Notes: ND: not determined; LOQ = 0.6 μg m^–3^.

b This study.

c ref 24.

As shown in Table 2, concentrations varied in a wide interval in both studies, which reflect the usual variability of atmospheric concentrations in a city, which is affected by a range of emission, transport, and meteorological parameters. The maximum values determined at UERJ, Maracanã (Table 2), as well as the outliers in Figure 1, were obtained on 28 June 28, 2023. The day was cloudy and cool and the atmospheric mixed layer was low. According to the report by the Municipal Department of Environment and Climate,^33^ the air quality in the city was in general “Moderate” and the 24 h mean PM_10_ concentrations were >50 μg m^–3^, the National Air Quality Standards established through the CONAMA Resolution N° 491.^34^

As da Silva et al.^24^ point out, HC concentrations are higher during the dry period (May-August). Anyway, total concentrations and individual values for most of the compounds were higher in 2013. The distribution of chemical groups is also different in the rain and wet seasons. In 2013, during the dry season, alkanes, alkenes, and aromatic compounds represented 77–78%, 7–10%, and 12–14%, respectively. During the 2016 Olympic Games, aromatic compounds at Saens Peña Square represented 10% of total determined HC.^31^ In 2023, these values were 85%, 9%, and 6%. Several aromatic compounds that were determined in the 2015 samples, were in concentrations < LOQ in all the 2015 samples: styrene, *n*-propylbenzene, (m + p)-ethyltoluenes, 1,3,5-trimethylbenzene, 1,2,4 and 1,2,3-trimethylbenzene. The reduction in aromatic compounds can also be attributed to the improvement in vehicular emissions. According to Siciliano et al.,^3^ the contribution of aromatic compounds to total exhaust emissions was approximately twice as much for the L5 vehicles as the L6.

Ozone forming potentials were evaluated using eq 1. HC, with median concentrations >0.6 μg m^–3^, were arranged in decreasing order of reactivity (OFP_i_), as shown in Figure 2. Then, the total average OFP was calculated using eq 2.

**Figure 2:**
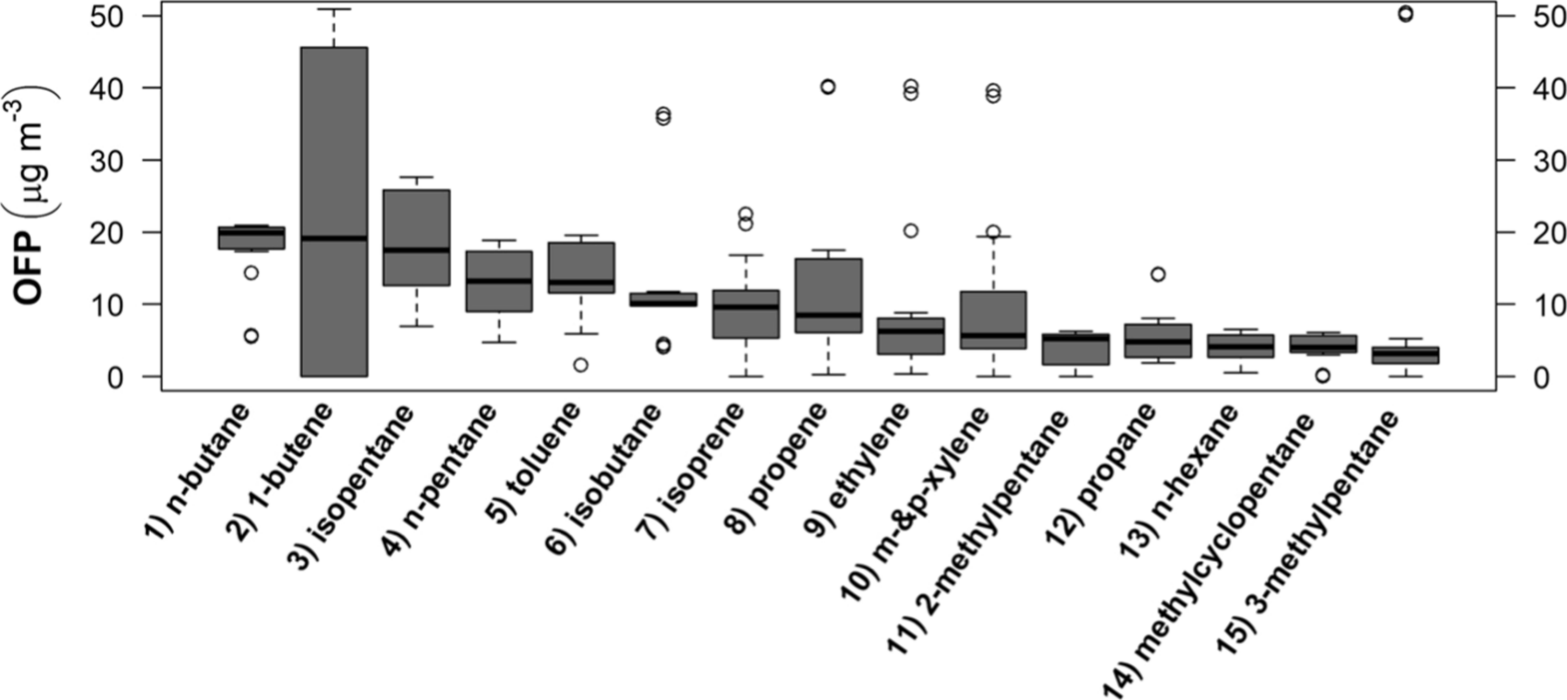
Ozone forming potentials (OFP_i_) calculated using the MIR scale for the top-15 most reactive C_2_–C_12_ compounds that were determined at the UERJ campus in May-July 2023.

The average median OFP (1.75 gO_3_/gHC) is lower than the values 2.78 to 2.51 gO_3_/gHC determined in 2015 at Saens Peña Square^24^ and about half the calculated value (3.57 gO_3_/gHC) for the District of Irajá.^32^ Calculated values for all of the samples are shown in Figure 3.

**Figure 3:**
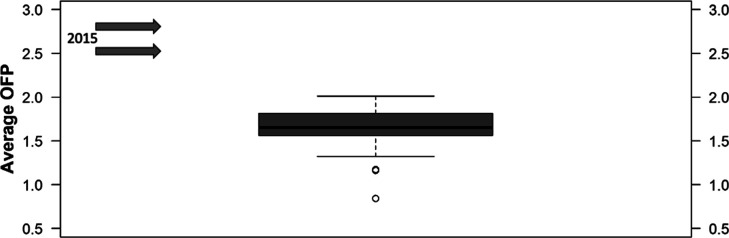
Average OFP calculated by the MIR scale for the C_2_–C_12_ compounds determined at the UERJ campus in May-July 2023. Values determined in 2015 (2.78–2.51 gO_3_/gHC) are shown for purposes of comparison.

As discussed by Zhang et al.,^35^ IR values are generally determined through model simulations and depend on the particular scenario, especially the NO_*x*_/VOC ratios. MIR values, as well other scales, such as Maximum Ozone Reactivity and Equal Benefit Incremental Reactivity do not necessarily apply to the city of Rio de Janeiro. For this reason, the values calculated in this study are approximate and are only used for purposes of comparison with data obtained previously in a similar scenario, where the same reactivity scale was employed. Clearly, emission changes in the past few years have resulted in a different composition of the urban atmosphere, especially in areas mostly impacted by vehicular emissions, and had led to a less reactive scenario due to the lower contribution of aromatic and alkenes to the emission mixture. In fact, chassis dynamometer studies have shown that the OFP for L6 vehicles is 0.015 gO_3_ km^–1^ in comparison to 0.088 gO_3_ km^–1^ for an L5 vehicle.^3^ It worth noting that the processes of formation and consumption of ozone depend on the composition of the VOC mixture but also on the VOC/NO_*x*_ ratio and meteorological conditions, in particular, temperature, humidity, and solar radiation.

The results of this study have some limitations. First, the number of samples is quite small and should be considered a preliminary study. Second, the study was limited to hydrocarbon compounds (C_2_–C_12_). The main reason for not including sampling and analysis of carbonyl compounds is that PROCONVE’s emission limits for phases L4-L6 are low and similar. Therefore, our objective was to evaluate the results of the more recent PROCONVE’s HC emission control policies. Third, a seasonal analysis was not performed because it was outside the scope of this study. But, our findings also have some strengths: the experimental method^1^ is a recent revision and improvement of Method TO-15A (US EPA); the traffic in the studied location is representative of the Rio de Janeiro fleet; and these are, to our knowledge, the first experimental data showing the contribution made by PROCONVE’s emission control policies to the total amount and speciation of C_2_–C_12_ HC.

## Conclusions

Since 1988, Brazilian vehicular emission standards have been revised and updated through several PROCONVE-L phases. NMHC emissions have been controlled since 2007, but it was after the L6 phase (launched in 2013), that a significant reduction of HC emissions was noted, especially of aromatic compounds. The renewal of the Brazilian fleet is slow since the average age of light-duty vehicles is 8–9 years.

In this study, the concentration, speciation, and ozone forming potentials (OFP_i_ and average OFP) of C_2_–C_12_ HC, collected, in 2023, in an urban area of the city of Rio de Janeiro that is characterized by vehicular emissions, were determined. The values were compared with previously published data, when there was a greater contribution of L4 and L5 vehicles to total emissions.

The results show a reduction in total concentrations, in particular of aromatic compounds, and in the average ozone forming potentials. Since samples were collected in the same seasonal period (dry season) and locations with similar emission features, it is clear that, despite an increase in the volume of traffic, the reduction in concentration and atmospheric reactivity can be attributed to the Brazilian Vehicular Emission Control Program and the improvement of the vehicular fleet. Since the L7 phase is currently in effect, a further reduction in NMHC can be expected in the future.

In the future, the calculation of a localized reactivity scale representative of the city of Rio de Janeiro, considering its particular environmental conditions, could be an important contribution to emission management and ozone control.

## Supplementary Material



## Notes

Map of the city of Rio de Janeiro and the studied area (UERJ, Maracanã) (PDF)
