# Supplementary material for: The Effects of Reducing Vehicular Emissions in Atmospheric Hydrocarbon Concentrations in Rio de Janeiro, Brazil
Source: ACS Omega. 2025 Nov 14;10(46):56183–90. doi: 10.1021/acsomega.5c07885 (PMC12658819; doi:10.1021/acsomega.5c07885)
Supplement: Supplementary file 1 [file ao5c07885_si_001.pdf]

The effects of reducing vehicular emissions in atmospheric hydrocarbon concentrations in Rio de Janeiro, Brazil

Margarida Maria Sartori Tavares[1], Rennan Guedes Carneiro[2], Graciela Arbilla[2*], Cleyton Martins da Silva[3], Sergio Machado Corrêa[1]

[1]Universidade do Estado do Rio de Janeiro, Resende, RJ, 27537-000, Brazil

[2]Universidade Federal do Rio de Janeiro, Instituto de Química, Rio de Janeiro, RJ, 21941-909, Brazil

[3]Universidade Veiga de Almeida, Campus Tijuca, Rio de Janeiro, RJ, 20271-020, Brazil

*Corresponding author: graciela@iq.ufrj.br

Supporting Information

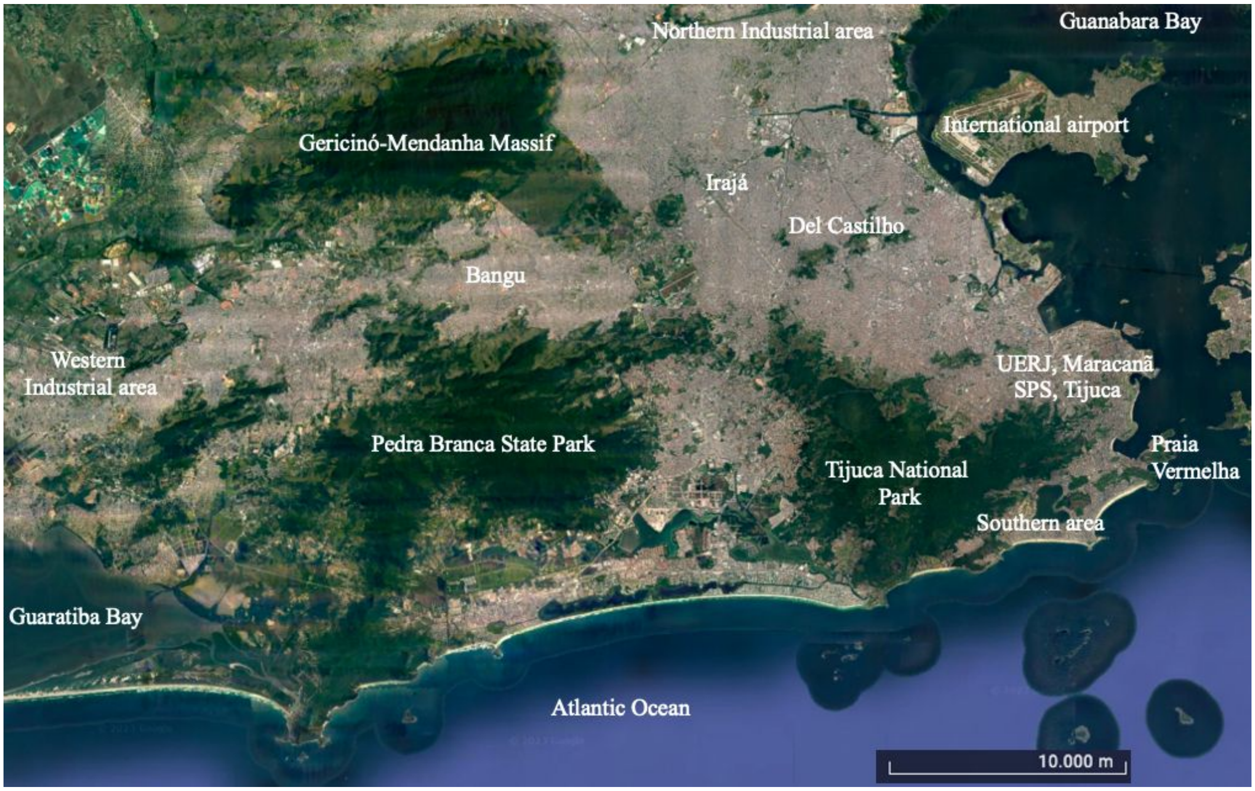

Figure S1. Map of the city of Rio de Janeiro and the studied area (UERJ, Maracanã). Other locations cited in the manuscript are also shown for reference purposes: Del Castilho, Irajá, Bangu, Saens Pena Square (SPS, Tijuca) and the industrial areas.
